# Cardiotoxicity of Microplastics: An Emerging Cardiovascular Risk Factor

**DOI:** 10.2174/011573403X366030250404105925

**Published:** 2025-04-10

**Authors:** Bhanu Duggal, Ghanshyam Kumar

**Affiliations:** 1 Department of Cardiology, All India Institute of Medical Sciences, Rishikesh, 249203, Uttarakhand, India

**Keywords:** Microplastics, cardiotoxicity, cardiovascular diseases (CVDs), atherosclerosis, thrombosis, ecosystems

## Abstract

The widespread use of plastics and improper disposal have resulted in the ubiquity of microplastics in the environment, from uninhabited polar regions to terrestrial ecosystems. This ubiquity poses significant health concerns for our environment and health. Various *in vitro*, *in vivo*, and *ex vivo* studies have indicated microplastic toxicity in humans' respiratory, digestive, neurological, reproductive, and developmental health. Recent studies have pointed out that these microplastics also have cardiovascular toxicity. Cardiovascular diseases (CVDs) are the number one killer in the world, with over 20 million annual deaths worldwide. Hence, microplastics, as a potential risk factor for CVDs, can result in a significant increase in mortality and morbidity because almost everyone is currently exposed to microplastics. This perspective article explores the toxic effects of microplastics on cardiovascular human health. It focuses on various studies that have found microplastics from human arteries/cardiac tissues and their potential role in atherosclerosis and subsequent increases in myocardial infarction, stroke, and mortality. Studies reported the presence of various microplastics, such as polyethylene, polyvinyl chloride, polyamide, and polystyrene, in cardiac tissues and arteries (coronary, aorta, cerebral, and carotid). Studies have suggested a potential negative correlation between microplastics and cardiovascular health, with the presence or increased concentration of microplastics linked to greater severity of health issues. Still, a causal relationship is yet to be established. Future studies, such as cohorts, should focus on deciphering and establishing whether microplastics are a potential cardiovascular risk factor.

## 1 INTRODUCTION

CVDs are the leading cause of mortality worldwide, with over 20 million deaths annually [1]. Various factors that contribute to CVDs include age, gender, ethnicity, family history, hypertension, hyperlipidemia, diabetes, high salt intake, smoking, obesity, poor diet, stress, sedentary lifestyle, *etc*. Identification of risk factors with advancements in medical technology has significantly decreased mortality caused by various CVDs [2-4]. Therefore, it is essential to identify the risk factors of CVDs so that they can be controlled and help in reducing mortality and morbidity. Environmental pollution, such as PM exposure, can significantly elevate the risk of CVDs [4]. The unprecedented use of plastics and their degradation to microplastics have resulted in their ubiquity in the environment [5]. Because of their ubiquity, they have entered humans through various exposure routes (ingestion, inhalation, and dermal exposure) and can accumulate in the body, posing a severe health issue [6, 7]. In the least developed or developing countries, due to limited resources and ineffective waste management, environmental pollution,including microplastics, plays a key role in CVD mortality [4, 8]. This perspective article focuses on the potential toxic effects of microplastics on cardiovascular health by critically analyzing studies conducted to decipher the impact of microplastics on CVDs. Literature searches in various databases, such as PubMed, Web of Science, and Google Scholar, have been done to find papers on microplastics and their relation to human cardiovascular diseases, including their role in thrombosis and atherosclerotic plaque formation. We have included all the relevant papers available in this field.

## 2 MICROPLASTICS AND THEIR TYPES

Microplastic, first recognized in 2004, is defined as plastic fragments with a diameter of less than 5 mm. Because of its slow degradation rate, it can have a toxic effect on the environment and health [9]. Reports suggest its presence in drinking water and even the food we eat, such as salts, honey, seafood, and crop plants [7, 10, 11]. Due to their ubiquity, almost everyone is currently exposed to microplastics with varying degrees of exposure [12]. These microplastics are further classified into primary and secondary microplastics, with primary being intentionally manufactured (microbeads in cosmetics) and secondary being the result of degradation of larger plastics [13].

## 3 MICROPLASTICS IN HUMANS

Research based on animal and cell studies limits our understanding of the impact of microplastics on human cardiovascular health. Hence, it is imperative to study the relationship between microplastics directly on *ex vivo* human samples and its direct relation between exposure levels and various disease severity. Microplastic pollution has received widespread attention in recent years, with many studies conducted in the past five years to study the cytotoxic effects of microplastics on human health. Microplastics have been detected in various human organs, including the reproductive, respiratory, neurological, cardiovascular, and digestive system (gut), as well as in excreta and the placenta [7, 9, 14]. Oxidative stress and immune modulation are the two common mechanisms of disease pathogenesis of microplastics [9]. The differential impact of microplastics on these mechanisms depends on the type of microplastics, their exposure time, and dose [15, 16]. Microplastics, especially nanoplastics, might accumulate in sites of atherosclerosis, as the small particles can easily cross the gut barrier, and the absorption in the circulatory system of these particles increases with a decrease in their size [17]. Microplastics entering the circulatory system and accumulating in the cardiovascular system can have serious health consequences [7, 18].

## 4 ANIMAL STUDIES TO STUDY THE IMPACT OF MICROPLASTICS ON CARDIOVASCULAR HEALTH

Various studies in animal models have shown that microplastics can induce various cardiac impairments, such as abnormal heart rate, pericardial edema, and myocardial fibrosis [19]. Reports from various animal models, including mice, rats, and zebrafish, as well as *in vitro* studies on human red blood cells and venous blood, have indicated the toxic effects of microplastics on the cardiovascular system. These effects include hemolysis, blood coagulation, platelet aggregation, and valve abnormalities [18, 19]. Microplastic exposure was found to increase creatine kinase-MB and troponin I levels in Wister rats’ serum. It caused apoptosis and structural damage due to induced oxidative stress and activation of the fibrosis-related Wnt/β-catenin signaling pathway [20]. PE nanoplastics in zebrafish embryos promoted the development of pericardial edema, endothelial cell dysfunction, and thrombosis, and inhibited angiogenesis [21]. In apolipoprotein E-deficient (ApoE^−/−^) mice, exposure to PS by drinking water resulted in various upregulation and downregulation of genes, mostly impacting immune modulation pathways. These pathways resulted in a change in pro-inflammatory and atherosclerotic phenotype. The exposure resulted in a significant accumulation of lipids in the heart valves and indicated that exposure might lead to atherosclerotic lesion formation [22].

## 5 MICROPLASTICS FOUND IN CARDIAC TISSUES AND ARTERIES

**Fig. (1):**
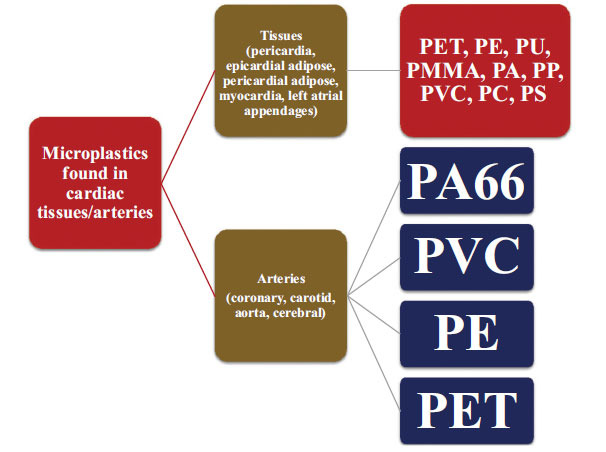
Types of microplastics found in cardiac tissues and arteries.

Studies indicate that microplastics have been found in various human cardiac tissues (pericardia, myocardia, left atrial appendages, pericardial adipose, epicardial adipose), and arteries, such as coronary, carotid, aorta, and cerebral (Fig. **1**). Six studies have reported the presence of microplastics in the human cardiovascular system, with three studies focused on linking their presence with disease severity [6, 7, 9, 12, 17, 23]. In ACS patients, microplastics, such as PE, PVC, PS, PA66, and PP, have been found, with the most prevalent being PE [9]. In patients with IS, MI, or deep vein thrombosis, microplastics, such as PA66, PVC, and PE, have been detected in the thrombotic arteries [7]. PE and PVC were detectable within the atherosclerotic carotid plaque in patients undergoing carotid endarterectomy [17]. Four types of microplastics, PET, PA66, PVC, and PE, were detected in coronary and carotid arteries having atherosclerotic plaques and in the aorta [6]. The presence of non-surgical plastics, such as PMMA, in cardiac tissue from patients undergoing cardiac surgery provides direct evidence of microplastics in human patients. Other microplastics found in cardiac tissues include PET, PU, PE, PP, PS, PA, PC, and PVC [23]. PE was also present at thrombotic sites in patients undergoing cardiovascular operations, suggesting its potential role in thrombosis [12].

## 6 MICROPLASTICS IN ATHEROSCLEROSIS AND THROMBOSIS

Due to their durability, microplastics can induce oxidative stress by generating reactive oxygen species, leading to DNA and cellular damage. They can also elicit immune-inflammatory responses, interfere with lipid metabolism (causing dyslipidemia), disrupt energy metabolism, and induce endothelial damage [7, 24-26].

These effects can contribute to the development of atherosclerosis in various ways. The inflammatory response elicited by microplastic can lead to an imbalance between pro-inflammatory and anti-inflammatory mechanisms/pathways, a crucial factor in atherosclerosis [9, 27]. Macrophages are the primary immune cells in cell-mediated immunity, and they are also the primary immune cells in atherosclerotic plaques; their interaction with microplastics can lead to various cytotoxic effects, such as the increased release of pro-inflammatory cytokines and impairment of lipid metabolism. Additionally, they can play an essential role in the progression of atherosclerosis and plaque instability [6, 28, 29]. Elevated microplastic concentrations have been found to be correlated with increased numbers of NK and B cells, suggesting that microplastic might promote atherosclerosis progression through immune cell activation [9]. The elevated microplastic concentration in arteries containing atherosclerotic plaques, compared to those without plaque, indicated its potential role in atherosclerosis [6]. Similarly, microplastics in arteries containing thrombus reaffirm the possible role of microplastics in thrombosis [12]. Microplastics are also the carriers of various POPs; when these POPS are transported through lipoprotein, they may get deposited in the plaque because of the lipid richness of the plaque [30].

Unstable plaques (composition includes cellular elements, lipids, calcium ions, and fibrin) lead to thrombosis [9]. Thrombus is a pathological structure formed within blood vessels and is the common cause of ischemic heart disease, stroke, and venous thromboembolism. Vascular endothelial damage and blood hypercoagulability play a crucial role in thrombus formation [12]. During the formation and enlargement of the thrombus, it could trap vascular contents and become the reservoir of microparticles, as circulating microplastics get trapped in developing thrombi, leading to the enlargement of the thrombus [7, 12]. These trapped microplastics may further enhance the deposition of platelets and fibrin in thrombi. Not only irregular microplastics but also spherical ones can accumulate in human thrombi [7].

## 7 MICROPLASTIC CONCENTRATION AND CVD SEVERITY

Studies that have tried to correlate the presence of microplastics and the CVD severity in humans are listed in Table **1**. Yang *et al*. reported that the average microplastic concentration was elevated in ACS compared to control patients (ACS: 161.65 μg per gram, control group: 100.13 μg per gram of blood); within ACS, patients with acute MI had a higher load of microplastics than those with unstable angina. The differential correlation between inflammatory cytokines and the type of microplastic indicates that the effect of microplastics depends on their physicochemical properties. Total microplastic content and the specific contents of PE, PVC, PS, and PP were positively correlated with the SYNTAX score, indicating that microplastics might play a role in coronary artery diseases [9]. Wang *et al*. reported an association between microplastic concentration and disease severity, with patients having higher microplastic concentrations showing increased severity of IS as assessed by the NIHSS (β of linear regression analysis = 7.72). In MI patients, the amount of microplastics varied from 49.3 μg/g in the left coronary artery to 234.3 μg/g in the right coronary artery [7]. A study by Marfella *et al*. on patients undergoing carotid endarterectomy found that those in whom MNPs were detected within the atheroma had a higher risk of a primary endpoint event compared to those in whom these microplastics were not detected. In 34 months of follow-up, patients with MNPs in the arteries had a higher risk of a composite of MI, stroke, or death than those in whom MNPs were not detected, as observed by a hazard ratio of 4.53. The positive correlation between inflammatory markers (interleukin-6, interleukin-18, TNF-α, and interleukin-1β) and the amount of MNPs reaffirmed the induction of pro-inflammatory pathways due to MNPs exposure (R^2^ varied from 0.319 to 0.497, with the corresponding slope values being positive) [17]. These results suggest that microplastics may pose a potential risk for CVDs by inducing immune and inflammatory responses, with their presence correlating with a decline in cardiovascular health.

## 8 LIMITATIONS

Studies conducted to decipher the role of microplastics on CVDs suffer from the following limitations:

1. Small sample size and lack of control in experiments conducted to study the potential risks of microplastics [9].
2. The lack of a representative sample in the studies makes generalization of results difficult for other populations and settings [17].
3. The cause-and-effect relationship between the presence and concentration of microplastics and the occurrence of thrombotic events and disease severity has yet to be established, as outcomes may also be influenced by other risk factors, including environmental pollutants, confounding variables, and the health status of the patients [7, 9, 17].

As listed above, the limitations of the current evidence on cardiotoxicity are that very few studies are available on this topic, and most studies have been conducted on *in vitro* (human cell lines) or animal models. Additionally, the studies that have tried to decipher the severity of CVDs with microplastic concentrations in humans are few, with small sample sizes, but they have indicated a possible linkage. There are also limitations due to the risk of contamination during sample collection and analysis.

## CONCLUSION

Microplastics have been found in various cardiac tissues and arteries. Studies indicate a possible linkage between microplastics and their role in atherosclerosis and thrombosis. Their presence has also been associated with an increase in CVD severity. A multi-centric study needs to be conducted with a more representative sample to study the dose-dependent and differential impact of various microplastics on CVD severity. A causal relationship between microplastic exposure and cardiovascular health is yet to be established. To establish this relation, cohort studies should be planned while considering confounding variables, such as other environmental pollutants, including PM2.5 and PM10 exposure, and other co-morbidities that can affect CVDs. Proper disposal of plastics should be done to minimize microplastic production, and environmentally friendly bioplastic use should be encouraged. Moreover, sensitization of people about the cytotoxicity of microplastics is also crucial in reducing their use and potential harmful effects.

## Figures and Tables

**Table 1:** Details of studies that have correlated the CVD severity with microplastic concentration.

**S. No.**	**Isolation of Microplastics**	**Total** **Sample Size**	**No. of Patients in which Microplastics were Detected**	**No. of Patients in which Microplastics were not Detected**	**CVDs Severity**	**References**
1	From coronary arteries, cerebral arteries, and deep veins of patients undergoing arterial or venous thrombectomy	30	24	6	The severity of stroke correlated with microplastic concentration (*p <* 0.05)	[7]
2	From blood samples of patients with complaints of chest pain and undergoing coronary angiography	101 (including 19 control)	101	0	High concentrations of microplastics correlated with high severity of CVDs (*p <* 0.0001)	[9]
3	From the carotid artery of patients undergoing carotid endarterectomy	257	150	107	Concentration of microplastics related to high occurrence of death, MI, and stroke (*p<*0.001)	[17]

## LIST OF ABBREVIATIONS

ACS: Acute Coronary Syndrome
CVDs: Cardiovascular Diseases
IS: Ischemic Stroke
MI: Myocardial Infarction
MNPs: Microplastics and Nanoplastics
NIHSS: National Institutes of Health Stroke Scale
PA: Polyamide
PC: Polycarbonate
PE: Polyethylene
PET: Polyethylene Terephthalate
PM: Particulate Matter
PMMA: Poly (Methyl Methacrylate)
POPs: Persistent Organic Pollutants
PP: Polypropylene
PS: Polystyrene
PU: Polyurethane
PVC: Polyvinyl Chloride
SYNTAX: Synergy between Percutaneous Coronary Intervention (PCI) with Taxus and Coronary Artery Bypass Surgery
